# Somatic and visceral nervous systems - an ancient duality

**DOI:** 10.1186/1741-7007-11-54

**Published:** 2013-04-30

**Authors:** Paola Bertucci, Detlev Arendt

**Affiliations:** 1 EMBL, Developmental Biology Unit, Meyerhofstrasse 1, Heidelbergs 69012, Germany

## Abstract

The vertebrate nervous system is deeply divided into ‘somatic’ and ‘visceral’ subsystems that respond to external and internal stimuli, respectively. Molecular characterization of neurons in different groups of mollusks by Nomaksteinsky and colleagues, published in this issue of *BMC Biology*, reveals that the viscero-somatic duality is evolutionarily ancient, predating Bilateria.

See research article: http://www.biomedcentral.com/1741-7007/11/53

## 

Starting with the work of the French physiologist Claude Bernard in the 19th century, vertebrates have been considered ‘dual entities’, composed of a ‘somatic’ and a ‘visceral’ animal responding to different environments: a *milieu extérieur* in which the organism is situated, and a *milieu intérieur* in which the tissue elements live [1]. Accordingly, somatic versus visceral nervous systems would mediate sensory-motor responses to the external and internal environment, respectively.

In recent years, the comparison of molecular fingerprints has been used for tracing the evolution of animal cell types. For example, it has been proposed that somato-motor neurons in vertebrates, insects, nematodes and annelids and branchio-motor neurons in ascidians and vertebrates are homologous cell types [2]. Nomaksteinsky *et al.*[3] have now used molecular fingerprinting to tackle a long-standing question in comparative neuroanatomy and neurophysiology, which is the evolutionary origin of the somatic versus visceral nervous system duality.

The vertebrate somatic nervous system involves somato-sensory neurons, located mostly in the dorsal root ganglia, ear and nose, perceiving environmental stimuli (primarily mechanical and olfactory, but also electromagnetic and thermal) and somato-motor neurons governing the locomotor response. The visceral nervous system, in turn, is composed of sensors for internal conditions and effectors controlling body homeostasis [4]. These viscero-sensory neurons are located in the blood vessels, airways or digestive tract monitoring the internal partial pressures of oxygen (pO2) and carbon dioxide (pCO2), blood pH, organ stretch and, last but not least, taste, and produce cardiovascular, respiratory and digestive responses.

The vertebrate somatic-visceral ‘duality’ is reflected by the differential usage of specific homeodomain transcription factors that drive cell-type specification. Subsets of somato-sensory neurons are specified by the POU domain factors *brn3a/b/c,* contributing to their development, axonal projection and survival [5]. In somato-motor neurons the EGH homeodomain transcription factor *hb9/mnx* is active, controlling specification, migration and axonal projection [6]. Finally, in almost all sensory and motor neurons of the vertebrate viscero-reflex-circuits the paired-like homeodomain transcription factor *phox2b* is present, likewise controlling specification, differentiation and axonal connectivity [7]. Previous studies indicated that the *brn3-hb9*-*phox2*-based differential specification of the somatic and visceral neurons is conserved outside vertebrates, at least to some degree. In *Caenorhabditis* and *Drosophila*, the *brn3* orthologs *unc86* and *acj6* are essential for specification and axonal targeting of the primary touch and olfactory somatic-sensory neurons, respectively [8,9]. *Drosophila hb9* specifies subsets of somato-motor neurons and interneurons and is required for the development of their projections. *phox2*, on the other hand, has been found in the motor neurons of the cerebral ganglion of the post-metamorphic ascidian *Ciona*, an invertebrate chordate, considered viscero-motor as they innervate the muscles of the branchial basket.

To elucidate the evolutionary origin of the viscero-somatic duality, Nomaksteinsky and co-workers have investigated several species of mollusks, which, together with annelids and other invertebrates, belong to the superphylum of Lophotrochozoa. Mollusks are an excellent choice, given that a huge body of electrophysiological, behavioral and anatomical studies revealed a rich diversity of visceral and somatic functions. The mollusk nervous system comprises ventral somatic (pedal) and lateral visceral chains of ganglia, connected by commissures and longitudinal connectives (Figure 1). While the pedal cord innervates the body and foot and mediates locomotion and escape behaviors, the visceral cord innervates the pharyngeal walls, salivary glands, mantle, kidneys, gonads, gills, liver, heart and gut, among others [10]. Nomaksteinsky *et al.* now show that *brn3* is expressed in clusters of mechano- and nociceptive sensory neurons (as identified by coexpression of the neuropeptide Sensorin A in the opisthobranch gastropod *Aplysia californica* (the California sea slug) and in the pulmonate snail *Lymnaea stagnalis*. In *Lymnaea*, *brn3* is co-expressed with *drgx* and *islet*, both of which are expressed in somato-sensory neurons in vertebrates, and with the vesicular glutamate transporter *vglut*, indicative of glutamatergic neurons as present in the murine dorsal root ganglia. The *brn3+/drgx+/islet+/vglut +* molecular fingerprint of vertebrate somato-sensory neurons thus appears to be conserved in mollusks. Likewise, the molecular fingerprint of vertebrate somatic-motor neurons (*hb9/mnx+/lhx3/4*+/acetylcholine+) [2] was detected in the pedal ganglia of *Lymnaea*, indicating evolutionary conservation. *hb9* was also found active in the pedal lobe of the subesophageal ganglionic mass of the cephalopod *Sepia officinalis*, in agreement with its locomotor function. Finally, Nomaksteinsky *et al.* detected expression of the visceral marker *phox2* in specific sets of previously described visceral neurons [10] in all groups, including the cephalopod-specific stellate ganglion, which contains postsynaptic motor neurons controlling the siphon, in line with the ancient ‘visceral’ nature of this structure (for respiration rather than propulsion).

**Figure 1.**
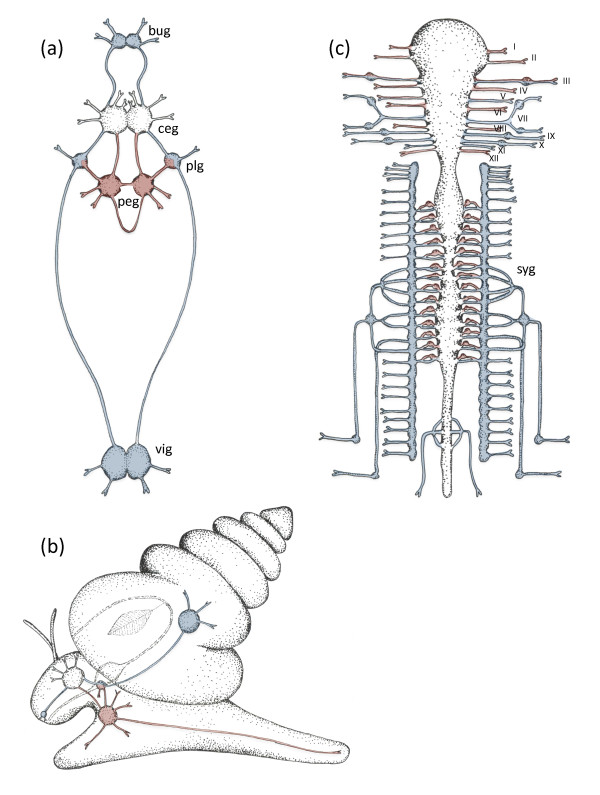
**The somatic (red) and visceral (blue) nervous systems in mollusks and vertebrates. **(**a**) In mollusks, the pedal cord interconnects the preoral cerebral ganglia (ceg) with the postoral pedal ganglia (peg). The visceral cord interconnects visceral ganglia (vig), pleural (plg) and buccal ganglia (bug), forming a second ring around the gut. (**b**) The same ganglia shown in an adult snail from a lateral view. (**c**) In vertebrates, roman numerals (I to XII) label the cranial nerves. syg, sympathetic ganglia.

Taken together, this is strong evidence that the somatic-visceral duality predates the bilaterian divergence. However, key questions remain. First, homology of somato-sensory and -motor neurons between mollusks and other bilaterians appears plausible given their conserved molecular fingerprints. Yet, the evidence is incomplete. In annelids and vertebrates, this homology is further supported by developmental data, showing that the respective neuron types arise from corresponding neurogenic columns [11]; in mollusks, the developmental origin of the respective neurons is unknown.

Regarding the significance of *phox2* expression in the visceral nervous system, any interpretation of the data in terms of a simple one-to-one cell type homology is difficult. Obviously, the set of vertebrate *phox2+* neurons is quite diverse, encompassing many different types of viscero-sensory as well as viscero-motor neurons, with divergent embryological origins, functions, innervation and target sites. Which of these would correspond to any of the mollusk neurons, which, among others, innervate mollusk-specific organs such as the mantle or the osphradia, special kinds of respiratory organ? Nomaksteinsky and colleagues refer to the concept of ‘deep homology’ (which implies the re-use of a conserved master regulator in evolution, leading to a similar cell fate) [12] to explain the common usage of *phox2* (and also *brn3*) in vertebrate versus mollusk visceral neurons. The shared expression of *phox2* would then merely reflect the conservation of a particular phenotypic trait that visceral neurons have in common and that is downstream of *phox2*, for example ‘to direct axonal projections towards somatic versus visceral targets’, as the authors propose. In their favor previous studies showed that the over-expression of *phox2* in non-visceral cells suffices to induce a visceral-motor axon phenotype [13]; and in *phox2* knockout mice visceral-sensory neurons change their axonal projections to those of somatic-sensory neurons [14]. Also, the authors argue that both the viscero-motor autonomic ganglia in vertebrates and the stellate ganglion in cephalopods are considered new characters (synapomorphies) and thus should not find a counterpart outside the respective groups; yet, they both express *phox2*. Once they evolved, the partaking neurons of these ganglia might have started to express *phox2* anew, thus becoming visceral and connecting to the pre-existing visceral circuits. However, this interpretation is problematic, too. It is hard to see what the highly diverse population of *phox2*+ neurons in vertebrates and mollusks has in common as a purely ‘visceral’ feature that could guide their proper interconnection not only as presynaptic neurons (that could be partially explained by the downstream expression of axonal guidance molecules, such as neural cell adhesion molecules) but also as postsynaptic neurons, targeted by pre-existing visceral neurons. Also, one would have to assume that ‘*de novo*’ evolving visceral neurons would start projecting to their new visceral targets from one generation to the next, once *phox2* is activated (by a cis-regulatory change), leaving little room for gradual evolution.

An alternative explanation might be the recently proposed ‘division of labor’ scenario [2]. This scenario assumes that neurons that partake in a given circuit have arisen by segregation of cellular functions to different sister cell types, which diverge but remain interconnected by axons. This concept allows for neuron types to be gradually relocated to remote locations during evolution. To stay with the example mentioned by the authors, the *phox2*+ first order (presynaptic) motor neurons of the palliovisceral ganglion and second order (postsynaptic) visceral-motor neurons of the stellate ganglion might have arisen from a single visceral neuron by cell type duplication. Then, the resulting post-ganglionic type would have been relocated to the periphery while the resulting pre-ganglionic neuron might have remained in the palliovisceral ganglion. This concept implies that there is not a simple one-to-one but rather a many-to-many homology-relationship between *phox2* neuron types of the circuits compared.

The work of Nomaksteinsky *et al.* raises additional interesting questions regarding the evolution of the somatic-visceral dualism. If both systems were in place in stem bilaterians, did they evolve independently or by duplication from a single, simple sensory-motor precursor circuit? And what were the most ancient sensory modalities and motor outputs of these two circuits, once they evolved? The most fascinating perspective comes from the fact that the urbilaterian ancestor was a marine animal as this has profound implications for what is regarded as external (somatic) and internal (visceral). In the marine world, the canonical viscero-sensory stimuli were clearly external: osmolarity, pH, pO2 and pCO2 reflect the quality of the surrounding water and thus inform the organism about the environment, the *milieu extérieur* (see above). Moreover, the chemical sense of taste, considered visceral by function and molecular identity, can also be regarded as ‘external’, as it informs about food. This would imply that both the ancient urbilaterian ‘visceral’ and ‘somatic’ circuits were in fact conveying information from the exterior. It appears that this environmental information has simply been ‘sorted’ into divergent circuits with regard to its (differential) downstream effect. For example, the somatic circuit processed all information relevant for locomotion (that is, mechanical stimuli and olfaction); part of the visceral circuit instead processed chemical information relevant for feeding and accordingly controlled the movement of the gut. Other parts of the ‘visceral’ circuit, however, might have integrated information regarding water quality, gas levels and pH into a - yet unknown - ancient effector system. Future work in simple marine invertebrates should elucidate these fascinating questions further.

## References

[B1] Romer AS The vertebrate as dual animal - somatic and visceral Evol Biol 1972 6 121 156

[B2] Arendt D The evolution of cell types in animals: emerging principles from molecular studies Nat Rev Genet 2008 9 868 882 10.1038/nrg2416 18927580

[B3] Nomaksteinsky M Kassabov S Chettouh Z Stoeklé HC Bonnaud L Fortin G Kandel ER Brunet JF Ancient origin of somatic and visceral neurons BMC Biol 2013 11 53 23631531 10.1186/1741-7007-11-53 PMC3660236

[B4] Blessing W The lower brainstem and bodily homeostasis Ann Neurol 1998 43 839

[B5] Zou M Li S Klein WH Xiang M Brn3a/Pou4f1 regulates dorsal root ganglion sensory neuron specification and axonal projection into the spinal cord Dev Biol 2012 364 114 127 10.1016/j.ydbio.2012.01.021 22326227 PMC3299823

[B6] Arber S Han B Mendelsohn M Smith M Jessell TM Sockanathan S Requirement for the homeobox gene Hb9 in the consolidation of motor neuron identity Neuron 1999 23 659 674 10.1016/S0896-6273(01)80026-X 10482234

[B7] Brunet JF Pattyn A Phox2 genes - from patterning to connectivity Curr Opin Genet Dev 2002 12 435 440 10.1016/S0959-437X(02)00322-2 12100889

[B8] Clyne PJ Certel SJ De Bruyne M Zaslavsky L Johnson WA Carlson JR The odor specificities of a subset of olfactory receptor neurons are governed by Acj6, a POU-domain transcription factor Neuron 1999 22 339 347 10.1016/S0896-6273(00)81094-6 10069339

[B9] Finney M Ruvkun G The unc-86 gene product couples cell-lineage and cell-identity in C. elegans Cell 1990 63 895 905 10.1016/0092-8674(90)90493-X 2257628

[B10] Bullock TH Horridge GA Structure and Function in the Nervous System of Invertebrates 1965 San Francisco, London: Freeman and company

[B11] Denes AS Jekely G Steinmetz PR Raible F Snyman H Prud’homme B Ferrier DE Balavoine G Arendt D Molecular architecture of annelid nerve cord supports common origin of nervous system centralization in bilateria Cell 2007 129 277 288 10.1016/j.cell.2007.02.040 17448990

[B12] Shubin N Tabin C Carroll S Deep homology and the origins of evolutionary novelty Nature 2009 457 818 823 10.1038/nature07891 19212399

[B13] Hirsch MR Glover JC Dufour HD Brunet JF Goridis C Forced expression of Phox2 homeodomain transcription factors induces a branchio-visceromotor axonal phenotype Dev Biol 2007 303 687 702 10.1016/j.ydbio.2006.12.006 17208219

[B14] D’Autreaux F Coppola E Hirsch MR Birchmeier C Brunet JF Homeoprotein Phox2b commands a somatic-to-visceral switch in cranial sensory pathways Proc Natl Acad Sci USA 2011 108 20018 20023 10.1073/pnas.1110416108 22128334 PMC3250195

